# Comprehensive diagnosis of PCDD/F emission from three hazardous waste incinerators

**DOI:** 10.1098/rsos.172056

**Published:** 2018-07-11

**Authors:** Xuan Cao, Longjie Ji, Xiaoqing Lin, William R. Stevens, Minghui Tang, Fanjie Shang, Shaofu Tang, Shengyong Lu

**Affiliations:** 1 State Key Laboratory of Clean Energy Utilization, Zhejiang University, Hangzhou 310027, People's Republic of China; 2 Zhejiang Fuchunjiang Environmental Technology Research Co. Ltd, Hangzhou 311401, People's Republic of China; 3 National Engineering Laboratory for Site Remediation Technologies, Beijing Construction Engineering Group Environmental Remediation Co. Ltd, Beijing 100015, People's Republic of China; 4 College of Health Sciences, Kentucky Christian University, Grayson, KY 41143, USA

**Keywords:** hazardous waste incinerator, start-up, normal operation, PCDD/F, memory effect, PCDD/F I-TEQ indicator

## Abstract

Comprehensive diagnosis of polychlorinated dibenzo-*p*-dioxin and dibenzofuran (PCDD/F) emissions was systematically conducted on three hazardous waste incinerators (HWIs). Results indicated that PCDD/F mainly existed in the solid phase before the bag filter. This was especially true for higher chlorinated dioxin and furan congeners (hexa-, hepta- and octa-). The aged bag filters tended to increase the gas-phase PCDD/F. Emissions also increased due to PCDD/F desorption from circulated scrubbing solution and plastic packing media used in the wet scrubber. The PCDD/F concentrations were elevated during the start-up process, reaching up to 5.4 times higher than those measured during the normal operating period. The ratios of PCDFs/PCDDs revealed that the surface-catalysed de novo synthesis was the dominant pathway of PCDD/F formation. Installation of more efficient fabric filters, intermittent replacement of circulated scrubbing solution will result in reduced PCDD/F emission. Additionally, 2,3,4,7,8-PeCDF correlated well with the international toxic equivalent quantity (I-TEQ) value, which suggests that 2,3,4,7,8-PeCDF could act as an I-TEQ indicator.

## 1. Introduction

In China, the amount of hazardous wastes (HWs) generated has sharply increased from 8.3 to 39.8 million tons from 2000 to 2015 [1,2]. According to United States Environmental Protection Agency, HWs pose substantial or potential threats to public health and the environment because of their ignitability, reactivity, corrosivity and toxicity. Therefore, the safe disposal of HWs has attracted wide attention among the public [3]. Several advanced techniques have been developed to treat HWs, including incineration, pyrolysis, safety landfill, solidification and biological treatment [4,5]. Among these, incineration has been regarded as a promising waste treatment technology due to its multiple advantages, such as energy recovery, volume reduction and chemical-toxicity destruction [6,7].

However, incineration will inevitably emit organic pollutants to the ambient atmosphere [8]. Polychlorinated dibenzo-*p*-dioxin and dibenzofuran (PCDD/F) emissions have attracted wide attention due to their highly toxic properties and persistence in the environment [9,10], because it has been reported that PCDD/F emission was present in the HW incineration process [11,12]. In addition, Gao *et al*. [13] investigated 14 domestic-made medical waste incinerators (MWIs) in China, of which nine were located along the eastern coast of China (Jiangsu, Shangdong and Liaoning Province), four were located in the remote areas (Sichuan, Yunan and Heilongjiang Province) and one was located in the central area (Henan Province). The results revealed that only two of 14 MWIs were operating below the European Union directive emission limit (0.1 ng international toxic equivalent quantity (I-TEQ) Nm^−3^), and a total amount of 4.87 g I-TEQ of PCDD/F was released to the atmosphere from MWIs in 2006. Chen *et al*. [14] revealed that PCDD/F emissions from hazardous waste incinerators (HWIs) were relatively lower than the values of MWIs.

The current PCDD/F emission standard of exhaust gas from HWIs is 0.5 ng I-TEQ Nm^−3^ in China. However, non-stationary combustion (including start-up and shutdown processes) conditions will increase PCDD/F emissions [15–17]. Lin *et al*. [6] found that the PCDD/F emissions during start-up were greater than those measured during normal operations. Wang *et al*. [18] found that the start-up phases accounted for 28% of the PCDD/F emissions from MWIs in a year which contained three start-up/shutdown cycles. This does not consider memory effects, which will result in increased PCDD/F concentrations at stack [19].

To correctly evaluate the exposures and potential health risks from incinerators, it is crucial to quantitatively determine emissions over a range of realistic operating conditions [16,17]. Additionally, a comprehensive diagnostic investigation, which seeks to compare PCDD/F emission levels at different functional devices of an incinerator facility simultaneously, is also needed to identify how different devices affect the PCDD/F emission levels [12]. To our knowledge, there is no such study of PCDD/F emission from HWIs. Previous results from municipal solid waste incinerators (MSWIs) showed the combustion chamber emitted little PCDD/F, compared with the PCDD/F emission from the boiler outlet [20]. Mariani *et al*. [21] found that PCDD/F emissions rose steeply after the boiler in a grate-type incinerator. A weak relationship was found between dioxin emissions and secondary chamber temperature [22].

Our investigations, conducted in 2014, feature simultaneous sampling of PCDD/F from three typical commercial-scale HWIs (HWI-A, HWI-B and HWI-C). Especially, a comprehensive diagnosis was conducted to explore the relationship between the air pollution control devices (APCDs) and PCDD/F emissions by measuring the PCDD/F concentrations, vapour/solid partitioning at the inlet and outlet of bag filters and the wet scrubber. Moreover, the PCDD/F contribution during start-up (HWI-B1) and normal operation (HWI-B2) are discussed. This study assists in our further understanding of the PCDD/F enhancement areas and in taking effective measures for controlling PCDD/Fs.

## 2. Material and methods

### 2.1 Hazardous waste incineration system

**Figure 1.**
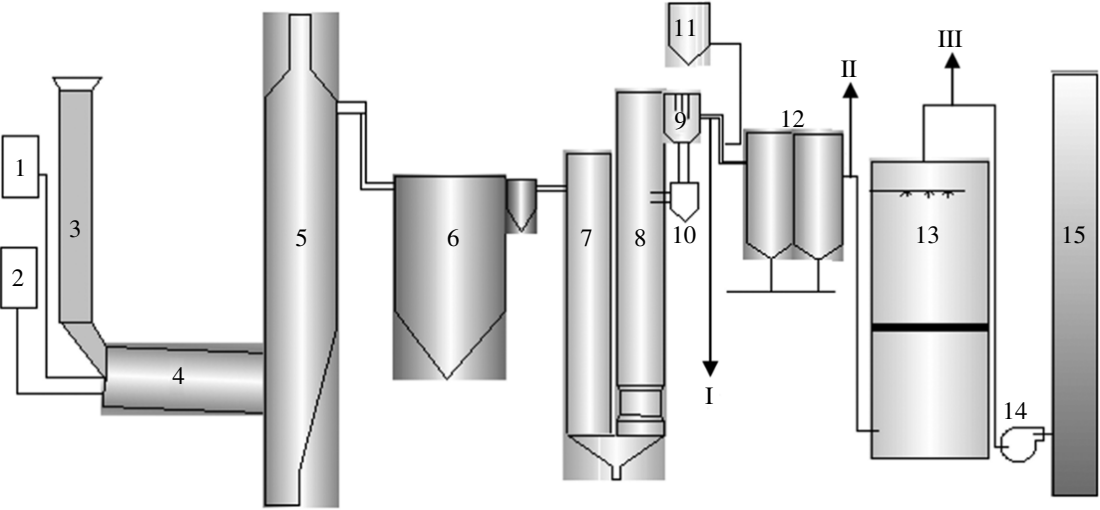
Schematic diagram and sampling sites of the investigated HWIs: (1) hazardous wastes; (2) air; (3) feeder; (4) rotary kiln; (5) secondary combustion chamber (outlet temperature: 1000–1100°C); (6) boiler (outlet temperature: 500–600°C); (7) quenching tower; (8) neutralization tower; (9) lime recycle device; (10) lime chamber; (11) activated carbon chamber; (12) bag filter; (13) wet scrubber; (14) fan; (15) stack. Sampling points: (I) quenching tower outlet (200–250°C); (II) bag filter outlet (180–220°C); (III) wet scrubber outlet (100–125°C).

The main constituents of a HW incineration system, together with their engineering characteristics have been described here. All three HWIs investigated here have the same structure and processes, featuring a two-stage combustion system, including a rotary kiln and a vertical secondary combustion chamber (figure 1). During normal combustion, HW was continuously fed into the rotary kiln with continuous rolling and mixing. HW was burned in the rotary kiln. However, due to limited oxygen, combustion was poor, resulting in many organic pollutants as products of incomplete combustion. Many of these organic pollutants are burned in a secondary combustion chamber where high temperatures (greater than 1100°C), long residence times (greater than 2 s) and abundant oxygen (excess air coefficient greater than 1.5) optimize the combustion efficiency of the system. The flue gas was then successively cooled by heat exchange in a boiler and a quench tower. Various pollutants in the flue gas are removed by the APCDs, including a dry lime scrubbing system, activated carbon (AC) injection system, fabric filter and scrubbing tower. AC powder is used to adsorb gaseous phase PCDD/F, and bag filters are used to remove particulate PCDD/F and AC [23]. Wet scrubbers are also effective in removing particulate-bound PCDD/F, which improves the potential reduction of total PCDD/F emissions [24].

The HW burned in the three involved incinerators mainly consisted of industrial HW and medical waste. A subset of relevant HWI operating parameters is given in table 1. The amount of AC added is in the normal range (50–300 mg Nm^−3^) and the amount of lime added is determined by the actual acid level in the neutralization tower. HWI-B was chosen to investigate the emission characteristics under start-up and normal operation conditions. The start-up procedure could be basically divided into two steps: (1) diesel oil as an auxiliary fuel burned with a maximum fuel feed rate until the suitable temperature was reached for incineration; (2) HW was input and the feed rate was increased until design capacity [16]. Flue gas was sampled at different sampling points during step 2 (denoted as HWI-B1), simultaneously. The incinerator reached steady operation after 30 h with a temperature ranging from 1100 to 1200°C in the secondary combustion chamber. The normal operation process of HWI-B was denoted as HWI-B2.

**Table 1.** Relevant operating parameters and sample time of three typical domestic hazardous waste incinerators.

operating parameters	HWI-A	HWI-B	HWI-C
location (China)	Shanghai	Hangzhou	Fuzhou
installed year	2012	2009	2011
nominal disposal capacity (t d^−1^)	80	50	30
flue gas flow (Nm^3^ h^−1^)	50 000	30 000	20 000
activated carbon added (mg Nm^−3^)	120	150	150
lime added (mg Nm^−3^)	3200	3500	3500
sample redundancy	triplicate	triplicate	triplicate

### 2.2 Sampling procedure and PCDD/F analysis

There were three sampling points in each HWI: quenching tower outlet, bag filter outlet and wet scrubber outlet, as shown in figure 1. For convenience, the quenching tower outlet and bag filter outlet are also referred to as bag filter inlet and wet scrubber inlet, respectively. All flue gas samples were simultaneously collected with isokinetic samplers (KNJ, Korea) according to US EPA 23a [25]. The sample collection component included a glass fibre filter followed by a sorbent (XAD-2 resin) module. The PCDD/F adhered to glass fibre filters and rinsed from the sampling probe thereafter was defined as solid-phase PCDD/F, while the rest adsorbed in XAD-2 resin was defined as vapour-phase PCDD/F; these two recovered samples were analysed separately by our accredited laboratory, which specializes in PCDD/F sampling and analysis. All samples were pretreated sequentially by soxhlet extraction, sulphuric acid wash and clean-up procedures. ^13^C-labelled EDF-4054, ^13^C-labelled EDF-4053 and ^13^C-labelled EDF-4055 were added before sampling of flue gas, extraction and purification, respectively. All standard solutions of PCDD/F were purchased from Cambridge Isotope Laboratory (Andover, MA, USA). The analysis was performed by high-resolution gas chromatography with high-resolution mass spectrometry (HRGC/HRMS) (JMS-800D, JEOL, Japan). A capillary column (DB-5MS, 60 m × 0.25 mm × 0.25 µm) was applied to separate the seventeen 2,3,7,8-substituted PCDD/F congeners and subsequently their concentrations were determined by mass spectrometry. The average recoveries of standards for PCDD/F range from 55% to 110%, which are all within the acceptable 25% to 130% range. All derived PCDD/F concentrations in flue gas were normalized to dry gas with respect to 273 K, 1013 hPa, and corrected for 11% oxygen content.

## 3. Results and discussion

### 3.1 PCDD/Fs at different sampling points

The average PCDD/F I-TEQ values of three HWIs during start-up and normal operation are summarized in table 2. I-TEQ emitted from stacks ranged from 0.039 to 0.53 ng I-TEQ Nm^−3^ during normal operation (HWI-A, HWI-B2 and HWI-C). These results were comparable to most of MWIs (0.08–0.5 ng I-TEQ Nm^−3^) reported by Gao *et al*. [13] The I-TEQ levels were also relatively lower than those of MSWIs (0.04–2.46 ng I-TEQ Nm^−3^) reported by Ni *et al*. [26]. Lower emission values could be a result of high temperature in the secondary combustion chamber, which caused further combustion of unburned particles or gases from the rotary kiln. The precursors of PCDD/F were burned out to avoid heterogeneous catalytic formation in low-temperature windows [27]. In addition, the HWIs were equipped with a quenching tower, which would effectively reduce the PCDD/F formation by quickly avoiding the optimum re-synthesis temperature window (250–400°C) [6,28,29].

**Table 2.** PCDD/F I-TEQ (ng I-TEQ Nm^−3^) in the three hazardous waste incinerators. I-TEQ were converted to dry normal conditions and 11% O_2_. The detection limit for I-TEQ is 0.001 ng I-TEQ Nm^−3^ at signal-to-noise ratio = 3.

	year installed	quenching tower outlet	bag filter outlet	wet scrubber outlet
HWI-A	2012	0.019 ± 0.002	0.242 ± 0.017	0.039 ± 0.003
HWI-B1	2009	5.81 ± 1.41	3.82 ± 0.43	2.85 ± 0.35
HWI-B2	2009	0.45 ± 0.08	1.33 ± 0.19	0.53 ± 0.07
HWI-C	2011	19.1 ± 3.2	0.175 ± 0.021	0.252 ± 0.034

The I-TEQ in HWI-A were the lowest among the three HWIs, with only an average value of 0.039 ng I-TEQ Nm^−3^ from the stack. This is probably related to the service life of HWI-A, which, at the time of sampling, had been in operation for only 3 years. For newly built incinerators, the operation conditions of the furnace and exhaust gas cleaning system are relatively optimal, which will efficiently reduce PCDD/F emissions [30,31]. Additionally, differences in PCDD/F concentrations can be attributed in part to difference in waste composition, operating conditions and the removal efficiencies of APCDs [30,32–34].

The I-TEQ values at the bag filter exit were ten times higher than the inlet of bag filters in HWI-A and HWI-B2, contrary to the traditional opinion of removing PCDD/F by using bag filters [7,35,36]. This is presumably ascribed to the heterogeneous catalytic (precursor or de novo) formation of PCDD/F in the bag filters [37–39]. It is also reported that spraying of powdered AC promoted the formation of PCDD/F by acting as both a catalyst substrate and carbon source [40]. Subsequent studies have shown that the specific surface area, pore diameter and feed rate of AC influenced the adsorption of PCDD/F [35,41].

The I-TEQ decreased by 83.9%, 25.4% and 60.2% after flowing through the wet scrubber for HWI-A, B1 and B2, respectively. However, it increased by 44% (0.077 ng/ I-TEQ nm^−3^) for HWI-C. It is assumed that the extended lifetime of APCDs may lead to increased PCDD/F emissions. Hence, special attention should be paid to older APCDs in commercial-scale incinerators.

### 3.2 Vapour/solid partitioning of PCDD/Fs in HWI-B2 and HWI-C

**Figure 2.**
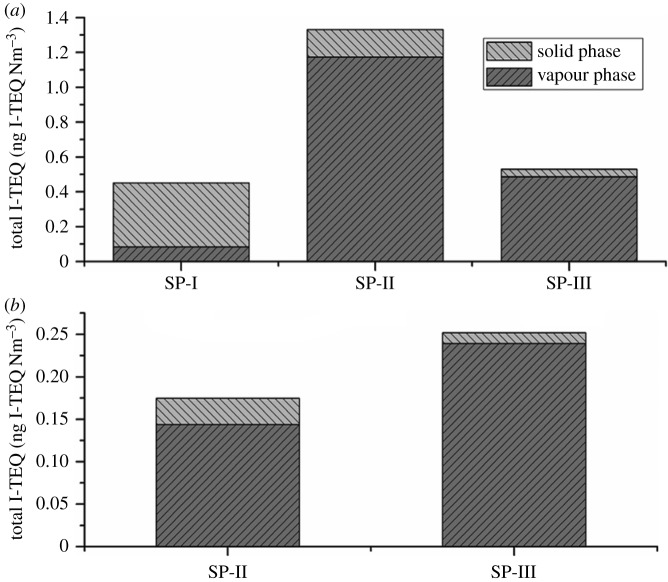
Vapour/solid partitioning of PCDD/F congener profiles in HWI-B2 (*a*) and HWI-C (*b*) (SP, sampling point).

PCDD/F emissions from incinerators are either gaseous or adsorbed to particulate matter (PM). The vapour/solid partitioning of PCDD/F varies with temperature, pressure and PM characteristics (including density, size or chemical composition) [42]. It is crucial to determine the form of PCDD/F present in flue gas for selecting the optimal design of controlling devices [43]. To explore the reasons for increased I-TEQ from bag filters and wet scrubbers, vapour/solid partitioning characteristics of PCDD/F in flue gas of HWI-B2 and HWI-C were further investigated (figure 2).

It was reported that PCDD/F mainly existed in the vapour phase at the outlet of the furnace section due to temperature [44,45]. Following gas flow to the bag filter, the solid-phase PCDD/F increased to 81.4% according to the I-TEQ at the bag filters’ inlet (HWI-B2). The large distribution difference could be due to the adsorption effect of porous fly ash, which could enrich the PCDD/F on the surface [35,42]. Another reason was that vapour pressures of those 17 congeners varied from 5 × 10^–10^ to 2 × 10^–7^ Pa at 25°C and increased as the chlorination level decreased [46]. Owing to the low vapour pressure of highly chlorinated PCDD/F, over 70% of those congeners are present in solid-phase PCDD/F [43]. This agreed with the results where highly chlorinated congeners accounted for 91.6 wt% of the total PCDD/F at the inlet of the bag filters in HWI-B2. After filtering, the partitioning of solid PCDD/F sharply decreased and vapour PCDD/F increased to 88.2% (HWI-B2) at the outlet of the bag filters [47].

**Figure 3.**
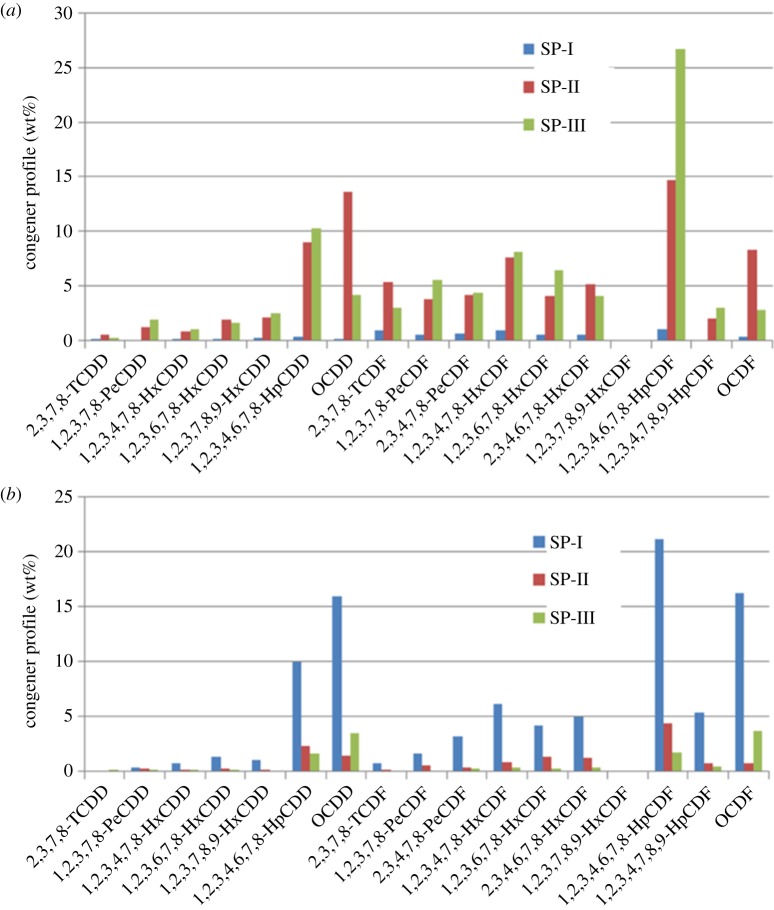
Distribution of the 17 vapour-phase (*a*) and solid-phase (*b*) 2,3,7,8-substituted dioxin congeners in HWI-B2 (SP, sampling point).

The acid gases and some small particles were removed by the wet scrubber. The removal efficiency of the wet scrubber in HWI-C was in line with Kreisz *et al*. [48], with an increasing concentration of PCDD/F after passing the wet scrubbers. The PCDD/F desorption from plastic packing media used in wet scrubbers is considered to be the main reason for PCDD/F enhancements [24]. Sam-Cwan *et al*. [49] revealed the PCDD/F enhancements were also caused by the circulated scrubbing solution, which contained as much as 20.9 ng I-TEQ L^−1^ (on average) of PCDD/F. They also found that PCDD/F tended to be diminished with increase in the proportions of AC mixed into the scrubbing solution. However, the PCDD/F removal efficiencies of the other three investigated operation conditions by the wet scrubber were in the range of 25.4 to 83.9%. These results are in agreement with previous data given by Vogg *et al*. [50]. Karademir & Korucu [51] studied the vapour- and solid-phase concentrations of the congeners after the wet scrubber and found that PCDD/F reduction was predominately related to the removal of solid-phase PCDD/F, whereas the vapour-phase PCDD/F concentrations did not change significantly. This explanation agrees with our analysis based on HWI-B2, where the removal efficiency for the solid phase (92%) was higher than that for the vapour phase (63%) after the wet scrubber. The partitioning of PCDD/F in the gas phase was further increased. The gas-phase congener profile is shown in figure 3. OCDD (15.9 wt%) was mainly presented in a solid phase before the bag filter. However, vapour-phase OCDD dominated the total PCDD/F at the outlet of the bag filter, accounting for 13.6 wt%. The dominating congener turned to vapour-phase 1,2,3,4,6,7,8-HpCDF at the outlet of the wet scrubber.

Hence, better practices regarding fabric filters and/or intermittent replacement of circulated scrubbing solution need to be adopted to reduce the concentration of PCDD/F in the exhaust gas [52–54]. For further reduction of the PCDD/F concentrations in flue gas, other coupling methods should be considered, such as nitrogen- or sulphur-containing inhibitors [55–57] and catalytic degradation technology [53,54].

### 3.3 I-TEQ during start-up (HWI-B1) and normal operation (HWI-B2)

Investigating the PCDD/F amounts and distributions during normal operation supplies the necessary baseline data of the HWI plants. Assessment and comparison of PCDD/F emissions during the start-up and normal operations are also useful to evaluate the effect on workers' health and the surrounding environment [6,51]. In this study, the start-up procedure could be basically divided into two steps, which have been mentioned above. The chlorine content in the waste played an important role in the PCDD/F emissions and congener profiles [58–61]. Wikström *et al*. [59] indicated that there was no correlation between the quantities of formed PCDD/F in the combustion process when the chlorine level in the fuel was below 1 wt%, but an increased formation rate was noted when the level of chlorine exceeded 1 wt%. The PCDD/F concentrations were relatively lower in the first step, due to the low chlorine content (less than 0.1 wt%) in diesel oil [51]. Thus, the discussion here mainly focuses on the second step (HWI-B1). The results showed numerous PCDD/Fs were formed due to the unstable combustion conditions characterized by an incomplete flue gas burnout during the start-up of HWIs, which was in line with MSWIs [15,51]. Especially, I-TEQ reached 2.85 ng I-TEQ Nm^−3^ (16.8 ng Nm^−3^) before the stack (HWI-B1). The PCDD/F emission level significantly deviated from the current emission standard (0.5 ng I-TEQ Nm^−3^) in China. The value was 5.4 (2.3) times higher than that of steady-state operation, which was 0.53 ng I-TEQ Nm^−3^ (7.15 ng Nm^−3^).

Memory effects, i.e. the PCDD/F or precursors adsorbed on the construction surface or fly ash are slowly desorbed into the flue gas, play an important role in the following period of normal operation conditions. Memory effects have been demonstrated in association with fly ash deposited on the boiler or APCDs, which act as reaction media and adsorption matrix for PCDD/F formation and subsequent release to the exhaust gas [37]. PCDD/F removal efficiency will decline gradually over time [19,62]. The excessive PCDD/F emission in HWI-B2 (0.53 ng I-TEQ Nm^−3^) could be attributed to this so-called memory effect. Moreover, other studies reported that the plastic wrapped materials used in wet scrubber systems markedly enhance the memory effect [63,64]. Drastic measures such as replacement of contacting materials and removing the residual fly ash deposited on the stack wall could effectively alleviate the memory effect [47,65,66].

### 3.4 PCDD/F congener profiles during start-up (HWI-B1) and normal operation (HWI-B2)

The details of the specific formation mechanism of PCDD/F still remain to be elucidated due to their low concentration and the difficulty of accurate ‘real-time’ analysis [67,68]. Explanations differ, yet it seems accepted that the main formation pathway is a catalytic process via precursor compounds and de novo synthesis [60,69]. During solid waste incineration, numerous factors have been reported to influence the PCDD/F formation, including residual carbon, O_2_, catalyst, sulphur, water, different forms of chlorine and nitrogen compounds (like ammonia and urea) [70].

**Figure 4.**
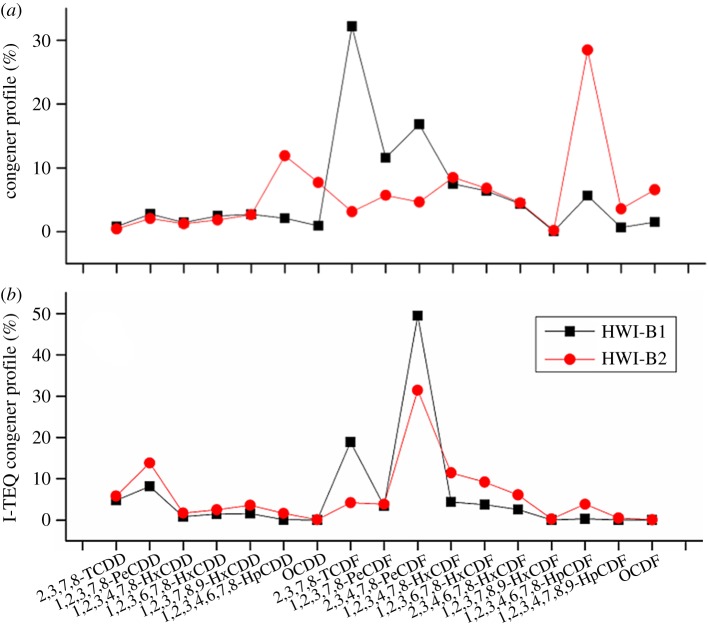
Characteristic of PCDD/F congener profiles at sampling point III during start-up and normal operation: (*a*) PCDD/F congener profiles and (*b*) I-TEQ congener profiles.

The mass and I-TEQ congener profiles of PCDD/F at the outlet of the wet scrubber are shown in figure 4. The most dominating congener during normal operation is 1,2,3,4,6,7,8-HpCDF (28.5 wt%) and during start-up operation 2,3,7,8-TCDF (32.2 wt%). However, Chen *et al*. [71] found that the most dominating congener during normal operation was 2,3,7,8-TCDF; during start-up it became 1,2,3,4,6,7,8-HpCDF when they investigated a medical waste incinerator. The average chlorination degree and PCDFs/PCDDs-partition ratio during start-up and normal operation may reveal mechanistic information about PCDD/F formation. During the start-up operation (HWI-B1), I-TEQ had sharply increased owing to an increase of incomplete combustion products. It was observed that the mass-averaged chlorination degree during start-up operation (64.2 wt% of low chlorination congeners) was less than the normal operation conditions (84 wt% of high chlorination congeners) at the outlet of the wet scrubber. The formation mechanism might be de novo synthesis, with the ratio of PCDFs/PCDDs being greater than 1 at different sampling points. Everaert & Baeyens [72] also indicated that de novo synthesis taking place between 200 and 400°C resulted in a much higher PCDFs/PCDDs ratio. In this study, the PCDFs/PCDDs ratio was 1.9 for HWI-B1 at the inlet of the bag filters, and lower than 2.3 for HWI-B2. The increase of the PCDFs/PCDDs ratio indicated that de novo synthesis was still the dominant pathway, whereas the precursor pathway favouring the formation of PCDDs also took place to some degree simultaneously.

2,3,4,7,8-PeCDF remains steadily at 40 ± 10% of the total I-TEQ in numerous thermal processes, including iron ore sintering and incineration [71]. In line with previous investigations on various matrices (flue gas, fly ash, ambient air or soil) in our research group [73–75] and elsewhere [13,16], 2,3,4,7,8-PeCDF was still the most abundant congener in TEQ concentration both during start-up (49.4%) and normal operation (31.4%) at the outlet of the wet scrubber. In addition, 2,3,7,8-TCDF and 1,2,3,4,7,8-HxCDF congeners changed significantly with respect to TEQ contribution when the combustion condition was different.

### 3.5 Evaluation of 2,3,4,7,8-PeCDF as toxic equivalent quantity indicators

**Figure 5.**
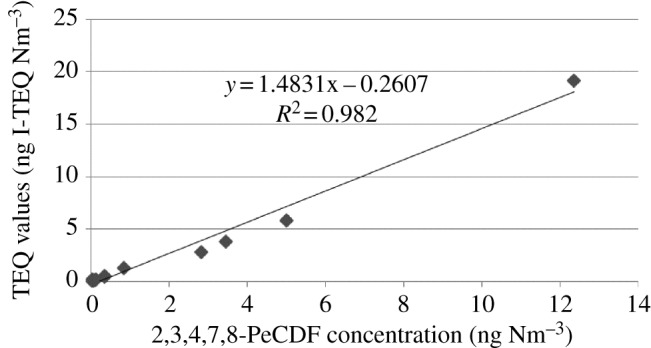
Correlation between 2,3,4,7,8-PeCDF concentration and TEQ values of PCDD/F.

It usually takes a few weeks to determine PCDD/F concentrations based on the chemical standard analysis method. Previous studies [76–78] have revealed that some organic indicators (so-called TEQ indicators) obtained a high correlation with the I-TEQ value of PCDD/F. This correlation could be used for an indirect online evaluation of the I-TEQ by some advanced laser mass spectrometric techniques, e.g. resonance-enhanced multi-photon ionization (REMPI) with time of flight mass spectrometry (TOFMS) [32]. Hence, screening of TEQ indicators was necessary for the real-time monitoring of PCDD/F emissions from waste incinerators. In this study, 2,3,4,7,8-PeCDF was the most important contributor to the I-TEQ value at the outlet of the wet scrubber, accounting for about 25.2%–55.4% (on average 39.3%) of three investigated HWIs. It was found that 2,3,4,7,8-PeCDF concentration was strongly correlated with I-TEQ (*R*^2^ = 0.982) as shown in figure 5. PeCDF has been proved as a TEQ indicator in a previous study based on a sensitive and robust vacuum ultraviolet (VUV) single-photoionization (SPI) ion trap (IT) time-of-flight mass spectrometer (VUV-SPI-IT-TOFMS) system [79]. Newly developed online monitoring technologies have made it possible to predict TEQ from homologue concentrations [79,80]. Therefore, 2,3,4,7,8-PeCDF could be considered as indicator isomers for the rapid determination of TEQ values, especially in long-term and real-time monitoring of one incinerator.

## 4. Conclusion

Among various safe disposal techniques, rotary kiln incineration of HWs has been extensively applied due to its marked advantages, such as complete decomposition and disinfection as well as significant volume and mass reduction. Comprehensive investigation of PCDD/F removal efficiencies in bag filters and wet scrubbers was performed for three HWIs. The ratio of PCDFs/PCDDs, and vapour/solid partitioning and congener profiles were explored, respectively. Both the absorptive memory effect (caused by aged bag filters and the wet scrubber) and the de novo-based memory effects (induced by the start-up process) leading to an increased PCDD/F emission have been observed. Results obtained indicate that the I-TEQ increased during the start-up process, reaching up to 5.4 times higher than those measured under normal operating conditions. The ratio of PCDFs/PCDDs revealed that the surface-catalysed de novo synthesis was the dominant pathway in the PCDD/F formation. The vapour/solid partitioning showed that PCDD/F mainly existed in the particulate phase before the bag filters, especially for the higher chlorinated ones. The bag filters could effectively remove solid-bound PCDD/F, but sometimes tend to increase the vapour-phase PCDD/F. PCDD/F desorption from plastic packing media or circulated scrubbing solution resulted in enhanced emission from the wet scrubber. More attention should be paid to these areas by applying more efficient fabric filters, intermittent dust removal and so on. In addition, 2,3,4,7,8-PeCDF, acting as an I-TEQ indicator, was still the most important contributor to the I-TEQ value and correlated well with calculated I-TEQ concentrations.

## Data accessibility

The data of this article are available at the Dryad Digital Repository: http://dx.doi.org/10.5061/dryad.qn54m. [81].
